# Editorial: Microbial OMICS, an asset to accelerate sustainability in agricultural and environmental microbiology

**DOI:** 10.3389/fgene.2024.1485895

**Published:** 2024-10-21

**Authors:** Adolphe Zézé, Mohamed Hijri

**Affiliations:** ^1^ Laboratoire de Microbiologie, Biotechnologies et Bioinformatique, Unité Mixte de Recherche et d’Innovation Sciences Agronomiques et Procédés de Transformation, Institut National Polytechnique Félix-Houphouet-Boigny, Yamoussoukro, Côte d’Ivoire; ^2^ Département de Sciences Biologiques, Institut de Recherche en Biologie Végétale, Université de Montréal, Montréal, QC, Canada; ^3^ African Genome Center, University Mohammed VI Polytechnic (UM6P), Ben Guerir, Morocco

**Keywords:** microbial omics, biomarkers, next-generation sequencing, ecosystems services, agricultural and environmental biotechnology

Microorganisms provide numerous ecosystem services to humans, allowing natural systems to benefit from a genetic reservoir essential for their fundamental functioning and sustainability (Rodrigues-Filho et al., 2023). They also play pivotal roles in the functioning of global ecosystems (Li et al., 2023; Liu et al., 2019). The advancement of microbial OMICS has enabled accurate elucidation of microbial functions across diverse ecosystems (Kaur et al., 2023), resulting in the identification and characterization of numerous provisioning services, biological processes, and supporting services (Beale et al., 2022). Moreover, microbial OMICS research has contributed to the advancement of applied biotechnologies and innovations in domains such as food security, agriculture, aquaculture, human health, animal health, and environmental protection (Su et al., 2024; Natnan et al., 2021; Chen et al., 2023; Goossens et al., 2022). The knowledge generated by microbial OMICS technologies, along with the development of related applied biotechnology, represents significant progress in sustainable agriculture and environmental management (Hijri, 2023).

**FIGURE 1:**
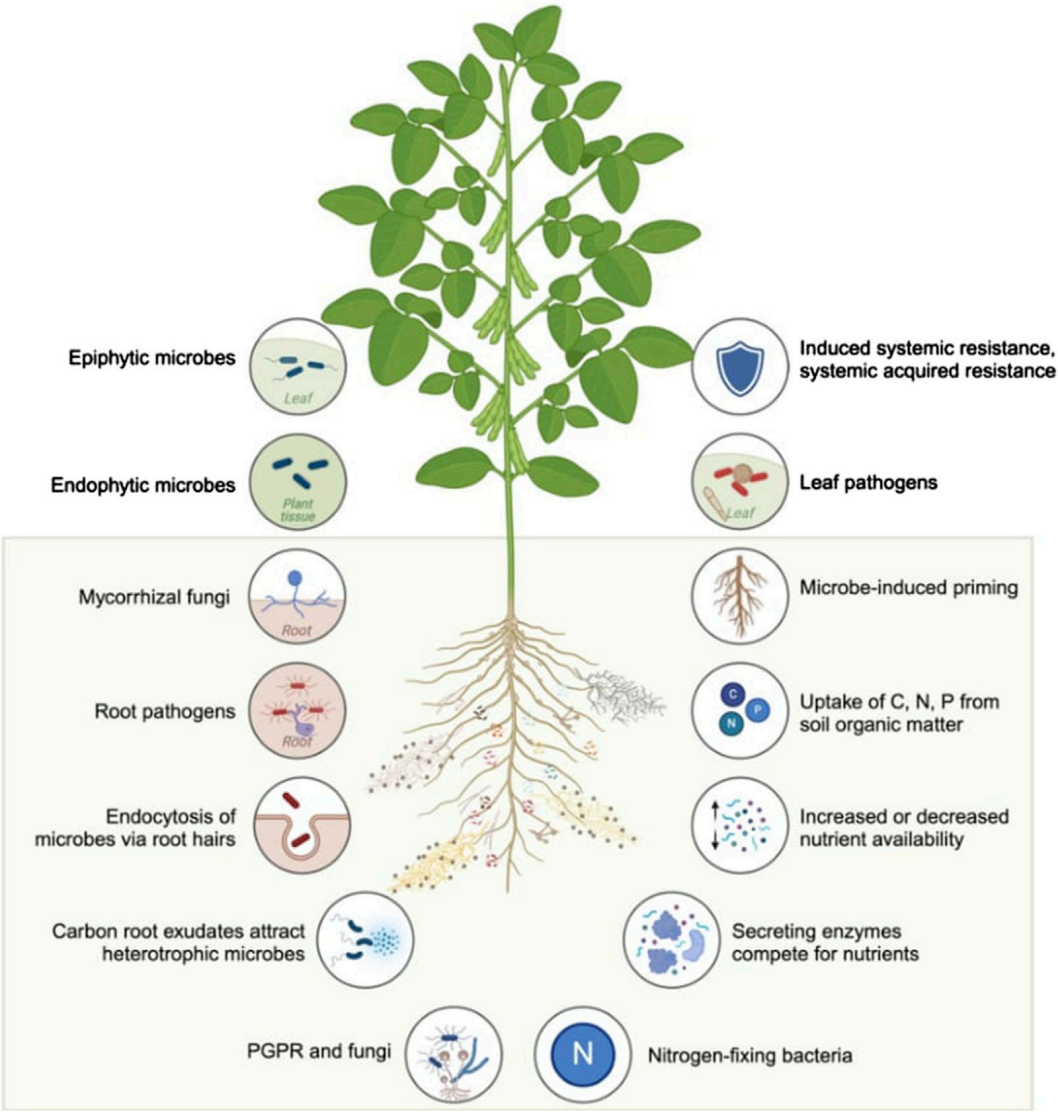
An overview of studies investigating plant-microbe interactions in soybean, a model crop, illustrating plant microbiota and microbial functions enabled by OMICS technologies. (Generated using BioRender.com).

The use of OMICS technologies enables the monitoring of soil health and productivity within agroecosystems. It has been reported that the utilization of specific microbes within agroecosystems, along with the type of soil management, can influence soil microbial community structure and function, consequently impacting soil health and crop productivity (Bertola et al., 2021). In a review, Adedayo and Babalola highlighted the significance of plant genomics in promoting the bioeconomy and the potential offered by advances in plant breeding techniques. Along with persisting challenges of underdevelopment and shifts in average weather conditions, the issue of food scarcity remains unresolved. Consequently, advances in crop production offer potential solutions to tackle these challenges. Furthermore, their review discussed the benefits of beneficial microbes in promoting crop growth and outlined the use of OMICS techniques to characterize plant-microbe interactions (Figure 1).

Using 16S rRNA gene metabarcoding targeting bacterial communities, Duan et al. demonstrated that the cultivation of *Morchella esculenta* in fallow paddy fields and drylands can alter the diversity of soil bacteria, affecting soil health and rice productivity. Their findings suggested that *M. esculenta* cultivation may enhance bioavailability of soil phosphorus and potassium in paddy fields, and potassium in dryland soil. *M. esculenta* cultivation had a modest impact on alpha diversity, and it influenced the abundance of certain genera of soil bacteria. Their functional annotation analysis indicated that *M. esculenta* cultivation might reduce methane production potential in paddy field soil and enhance nitrogen cycling in dryland soil. They performed a network analysis and correlation analysis, which revealed that *Gemmatimonas, Bryobacter*, and *Anaeromyxobacter* were key bacterial genera regulating soil chemical properties in paddy field soil under *M. esculenta* cultivation, while *Bryobacter, Bacillus, Streptomyces*, and *Paenarthrobacter* were key taxa associated with potassium accumulation in dryland soil.

Arunrat et al. compared the variability of soil bacterial communities in maize monoculture and the fallow phase of rotational shifting cultivation fields in Northern Thailand. They selected a continuous 5-year fallow field (CF-5Y) and a continuous 5-year maize cultivation field (M-5Y) with similar microclimate, topography, and duration of field activities. Soil samples were collected from the surface layer at both sites every 3 months for 1 year. Analysis of soil bacterial diversity and composition was conducted using 16S rRNA gene amplicon sequencing. They observed that CF-5Y soil maintained greater stability in bacterial richness and diversity across seasons than M-5Y soil. Notably, fertilization and tillage practices in M-5Y were found to enhance both the diversity and richness of soil bacteria. The study concluded that changes in soil bacterial diversity may result from multifactorial conditions such as land management practices, soil physicochemical properties, weather conditions, and vegetation cover (Arunrat et al.).

Another finding relates to how soil health and crop productivity can be impacted by organic amendment into plant-fungus intercropping systems. Duan et al. assessed the impact of bagasse amendment in a sugarcane - *Dictyophora indusiata* intercropping system on soil health through a field experiment comprising three treatments: bagasse amendment alone, (2) sugarcane amended with bagasse, and the control. Soil chemistry, soil bacterial and fungal diversity using amplicon sequencing, and metabolite composition were analyzed to elucidate the mechanisms underlying the effects of this intercropping system on soil properties. Soil chemistry analyses revealed higher levels of several soil nutrients such as nitrogen and phosphorus in the bagasse application compared to the control. Bacterial diversity was greater in the bagasse application than other treatments, while fungal diversity was lower in the bagasse-amended sugarcane than in other treatments. Soil metabolome analysis revealed significantly lower abundance of carbohydrate metabolites in the bagasse application compared to the control and the bagasse-amended sugarcane. They suggested that the sugarcane amended with bagasse can improve soil health in this intercropping system.

Two studies on microbial genome sequencing revealed new species. A *Bradyrhizobium*. isolated from polluted sediments of a lake in China was identified as a new free-living species *Bradyrhizobium roseum* (Zhang et al.). *B. roseum* displays considerable heterogeneity, exhibiting several functional distinctions from previously described *Bradyrhizobium* genomes. Another study reported a new endophytic fungus, *Cladosporium angulosum*, harboring N uptake-related genes with the potential to enhance plant growth (Yang et al.).

Amon et al. utilized 16S rRNA gene analyses to explore bacterial biogeography and ecosystem services in soils of Côte d’Ivoire. Within bacterial communities comprising 48 phyla, 92 classes, 152 orders, 356 families, and 1,234 genera in Côte d’Ivoire soils, a core bacteriobiome was identified. The distribution of the core genera, along with the 10 major phyla, was influenced by environmental factors including latitude, and soil pH, Al, and K. The predominant distribution pattern observed for the core bacteriobiome was vegetation-independent. Concerning predicted functions, all core bacterial taxa were implicated in assimilatory sulfate reduction, while atmospheric dinitrogen (N_2_) reduction was exclusively associated with the genus *Bradyrhizobium*.

Carroll et al. employed phylogenomic and comparative genomics to characterize the population structure and functional potential of 110 *Micrococcus* strains, comprising 104 publicly available genomes and six individuals isolated from South Africa. In terms of functional potential, genes for antimicrobial compounds, including macrolides, beta-lactams, and aminoglycosides (in 81, 61, and 44 of 110 genomes, respectively), were identified across *Micrococcus* genomes. Genome-wide analyses enabled Ma et al. to understand variation in *Mycoplasma gallisepticum*, one of the primary causative agents of chronic respiratory diseases in poultry. It was revealed that the intensity of *M. gallisepticum* biofilm formation strongly correlates with chronic infection, and strains with stronger biofilm-forming abilities exhibit reduced sensitivity to 17 tested antibiotics. Putative key genes associated with biofilm formation, identified through genome-wide analysis of two strains with contrasting biofilm formation, included ManB, oppA, oppD, PDH, eno, RelA, msbA, deoA, gapA, rpoS, Adhesin P1 precursor, S-adenosine methionine synthetase, and methionyl tRNA synthetase.

Two investigations underscored the significance of microbial OMICS in elucidating natural environmental microbiological processes. de Oliveira et al. utilized shotgun metagenomic sequencing data from water column samples to investigate the resistome and bacterial diversity of two small lakes in the Southern Pantanal region of Brazil. The Abobral lake displayed the highest diversity and abundance of antibiotic resistance genes, antibiotic resistance classes, phyla, and genera. RPOB2 was identified as the most abundant antibiotic resistance gene, and its associated resistance class was the most abundant class. Pseudomonadota emerged as the dominant phylum across all sites, with *Streptomyces* being the most prevalent genus. Ye et al. isolated a thermophilic *Bacillus subtilis* strain from nicotine waste that was resistant to nicotine and possessed high capability to degrade tobacco-derived organics. Whole-genome sequencing revealed that this *B. subtilis* strain also exhibited antibacterial properties, enabling its use in organic fertilizers capable of biological control.
